# Combining Intraoral and Face Scans for the Design and Fabrication of Computer-Assisted Design/Computer-Assisted Manufacturing (CAD/CAM) Polyether-Ether-Ketone (PEEK) Implant-Supported Bars for Maxillary Overdentures

**DOI:** 10.1155/2019/4274715

**Published:** 2019-08-22

**Authors:** Francesco Mangano, Carlo Mangano, Bidzina Margiani, Oleg Admakin

**Affiliations:** ^1^Lecturer, Department of Prevention and Communal Dentistry, Sechenov First Moscow State Medical University, 119992 Moscow, Russia; ^2^Professor and Lecturer, Department of Dental Sciences, Vita and Salute University San Raffaele, 20132 Milan, Italy; ^3^Professor and Head, Department of Prevention and Communal Dentistry, Sechenov First Moscow State Medical University, 119992 Moscow, Russia

## Abstract

**Purpose:**

To present a digital method that combines intraoral and face scanning for the computer-assisted design/computer-assisted manufacturing (CAD/CAM) fabrication of implant-supported bars for maxillary overdentures.

**Methods:**

Over a 2-year period, all patients presenting to a private dental clinic with a removable complete denture in the maxilla, seeking rehabilitation with implants, were considered for inclusion in this study. Inclusion criteria were fully edentulous maxilla, functional problems with the preexisting denture, opposing dentition, and sufficient bone volume to insert four implants. Exclusion criteria were age < 55 years, need for bone augmentation, uncompensated diabetes mellitus, immunocompromised status, radio- and/or chemotherapy, and previous treatment with oral and/or intravenous aminobisphosphonates. All patients were rehabilitated with a maxillary overdenture supported by a CAD/CAM polyether-ether-ketone (PEEK) implant-supported bar. The outcomes of the study were the passive fit/adaptation of the bar, the 1-year implant survival, and the success rates of the implant-supported overdentures.

**Results:**

15 patients (6 males, 9 females; mean age 68.8 ± 4.7 years) received 60 implants and were rehabilitated with a maxillary overdenture supported by a PEEK bar, designed and milled from an intraoral digital impression. The intraoral scans were integrated with face scans, in order to design each bar with all available patient data (soft tissues, prosthesis, implants, and face) in the correct spatial position. When testing the 3D-printed resin bar, 12 bars out of 15 (80%) had a perfect passive adaptation and fit; in contrast, 3 out of 15 (20%) did not have a sufficient passive fit or adaptation. No implants were lost, for a 1-year survival of 100% (60/60 surviving implants). However, some complications (two fixtures with peri-implantitis in the same patient and two repaired overdentures in two different patients) occurred. This determined a 1-year success rate of 80% for the implant-supported overdenture.

**Conclusions:**

In this study, the combination of intraoral and face scans allowed to successfully restore fully edentulous patients with maxillary overdentures supported by 4 implants and a CAD/CAM PEEK bar. Further studies are needed to confirm these outcomes.

## 1. Introduction

The digital revolution is changing the world of dentistry [1]. Intraoral scanners (IOSs) [2, 3], face scanners (FSs) [4, 5], and cone beam computed tomography (CBCT) [6] allow the dentist to capture three-dimensional (3D) information about the patient and, from such data acquisition, create virtual models of teeth, face, and bone bases. These data are then imported into specific computer-assisted design (CAD) software and superimposed upon each other in order to obtain the “virtual patient” [7, 8], the starting point for 3D surgical, prosthetic, and orthodontic planning. Within the CAD software, the dentist and dental technician plan the therapy and design a series of devices (surgical templates [9, 10], prostheses [11–13], and orthodontic devices [14]) to be used on patients. Finally, these devices are processed by appropriate computer-assisted manufacturing (CAM) software, milled or 3D printed, and are available for clinical use [15].

In the prosthetic field, the digital revolution has a strong impact because the dentist can capture optical impressions with IOS [2, 3, 11–13]; these impressions are used by the dental technician for the planning and hence the production of a whole series of fixed prosthetic restorations (inlays [12, 16], onlays [16], single crowns [17, 18], and bridges of up to 4 or 5 elements [19]). The literature now shows that all these applications are possible and represent a clinical reality [11]. Patients favor optical impressions, which have eliminated the need for conventional analog impressions with trays and materials (alginate, polyvinylsiloxane, and polyether) [2, 20, 21]. The optical impressions also eliminate the discomfort linked to the conventional analog impressions; they are easy to capture for the clinician (even in the presence of undercuts or dental implants), and they can be sent directly to the dental laboratory by e-mail, at no cost [2, 20]. The dental technician can view the impressions and immediately give feedback to the clinician, while the patient sits comfortably in the dental chair. Furthermore, the high quality of the 3D images derived from the optical impressions even makes the IOS useful as a marketing tool with patients.

Although IOSs are becoming widespread and have become a very useful tool for capturing impressions in partially edentulous patients [2, 11–19], the scientific literature does not seem to support their use in completely edentulous patients [22–24]. Numerous systematic reviews suggest that IOSs do not yet have adequate accuracy to allow CAD and thus the fabrication of full-arch-type restorations [22–24], particularly in patients with implants [23, 24]; in this, the distance between the implants seems to play a major role [25].

However, data emerging from these revisions stem from the analysis of previous clinical trials, in which first-generation IOSs were used [22–24]. The technological evolution is proceeding very fast, and the manufacturing companies release new hardware and software every month to improve the accuracy of their IOS; scientific literature has different times and struggles to follow. Furthermore, it must be emphasized that there are statistically significant differences in the accuracy of different IOSs, especially in scans of completely edentulous patients [3, 26]. Moreover, the restoration of the completely edentulous patient can take place with a fixed prosthesis supported by 6–8 implants [27, 28], such as with a bar-retained overdenture supported by 4 implants [29]; in the latter application, the implants are closer to each other, generally inserted into the anterior area of the maxilla, in which case the optical impression can be less difficult.

Recently, in fact, some clinical studies have shown that using the latest-generation IOS, it is possible to design and fabricate clinically precise CAD/CAM implant-supported bars [30, 31]. Today, this is possible and represents an important step forward in the field of digitalization of prosthetic procedures within the dental practice; it is in fact possible to plan the shape and volume of the bar according to the prosthetic spaces available [30, 31]. In this context, the acquisition of the patient's face via FS represents a further important development, not only to facilitate the modeling of the bar in relation to tissue volumes but also to present the case to the patient.

The aim of the present prospective clinical study is to present a digital method that combines intraoral and face scanning for the CAD/CAM fabrication of implant-supported bars for maxillary overdentures.

## 2. Materials and Methods

### 2.1. Patient Selection

Over a 2-year period (2017–2018), all patients presenting to a private dental clinic, and seeking prosthetic rehabilitation with implants, were considered for inclusion in this prospective clinical study. Inclusion criteria for enrollment in the study were (1) fully edentulous maxilla; (2) functional problems with the complete removable denture (e.g., lack of stability, discomfort due to the size of the prosthesis); (3) presence of opposing natural or artificial dentition in the antagonist arch; (4) sufficient bone volume to be able to insert four implants of standard diameter and length (at least 3.3 mm × 10 mm), suitable for supporting a bar, in the anterior maxilla; and (5) good general health status. Exclusion criteria for enrollment in this study were (1) age < 55 years; (2) previous bone augmentation techniques and/or regenerative bone procedures or need to proceed with them, in order to be able to insert dental implants; (3) uncompensated diabetes mellitus; (4) immunocompromised status; (5) radio- and/or chemotherapy; and (6) treatment with aminobisphosphonates (taken orally or parenterally). The patients who presented with the conditions listed in the inclusion criteria, and who did not have any of those listed in the exclusion criteria, were informed in detail about the possible therapeutic strategies (fixed prosthesis supported by 6–8 implants or bar-supported overdenture sustained by 4 implants) as well as their advantages and limitations. At the end of the informational interview, all patients who opted for rehabilitation with bar-retained overdentures were included in the present clinical study. Before starting the treatment, all the enrolled patients were informed of the importance of avoiding smoking, since smoke represents a risk factor for implant failure in the short and long term [32]. In addition, they received detailed information on the potential risks related to the implant treatment and signed an informed consent and an authorization for inclusion in the study. This study was approved by the Ethics Committee of Sechenov First Moscow State Medical University and was conducted in accordance with the principles set out in the 1975 Helsinki Declaration on clinical research involving humans, as revised in 2008.

### 2.2. Clinical and Laboratory Procedures

**Figure 1:**
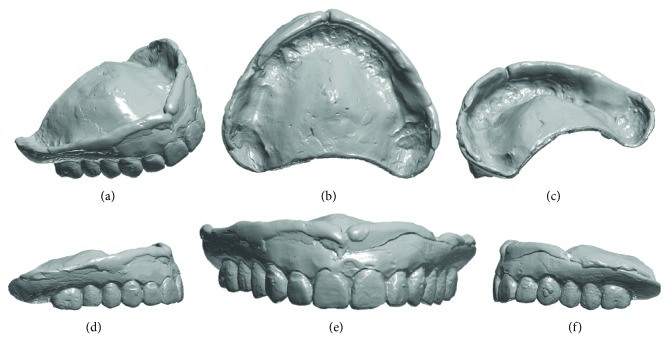
Extraoral scan of the preexisting complete removable denture, suitably relined with CS 3600® (Carestream Dental, Atlanta, GE, USA). (a) Anterior perspective view; (b) vision of the inner part in contact with the mucosal tissues; (c) posterior perspective view; (d) right side view; (e) front view; (f) left side view.

**Figure 2:**
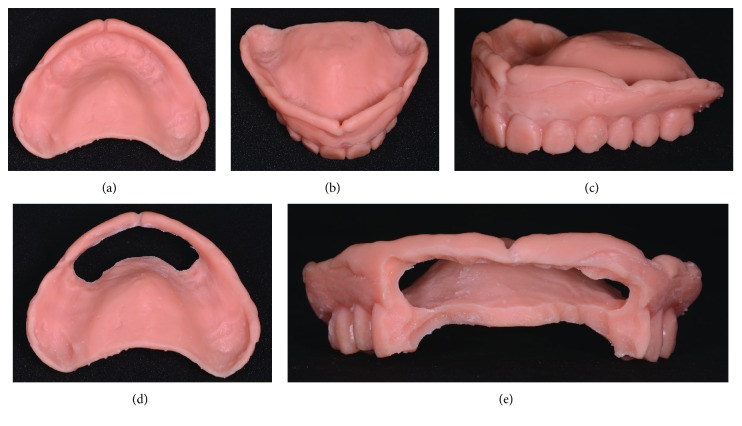
The copy of the preexisting complete removable denture, relined and extraorally scanned, is printed with a stereolithographic 3D printer (3500PD®, DWS, Thiene, Vicenza, Italy) and subsequently discarded and opened in the area of scanbodies. (a) Complete copy of the preexisting denture, internal view; (b) full copy of the preexisting denture, anterior view; (c) full copy of the preexisting denture, perspective view; (d) the copy of the preexisting denture discarded and opened in the anterior area, in correspondence with the emergencies of the scanbodies, internal view; (e) the copy of the preexisting denture in the anterior area, in correspondence with the emergencies of the scanbodies, frontal view.

**Figure 3:**
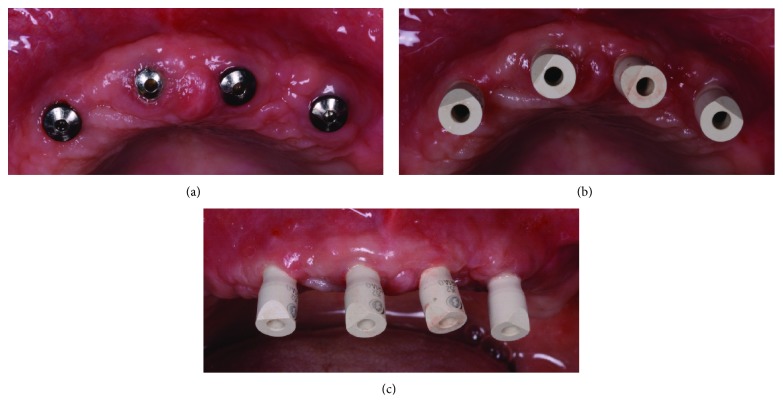
Intraoral scanning clinical images. (a) The implants before the removal of healing abutments; (b) scanbodies (BTSafe® scan abutments, BTK, Dueville, Vicenza, Italy) in position, occlusal view; (c) scanbodies in position, frontal view.

**Figure 4:**
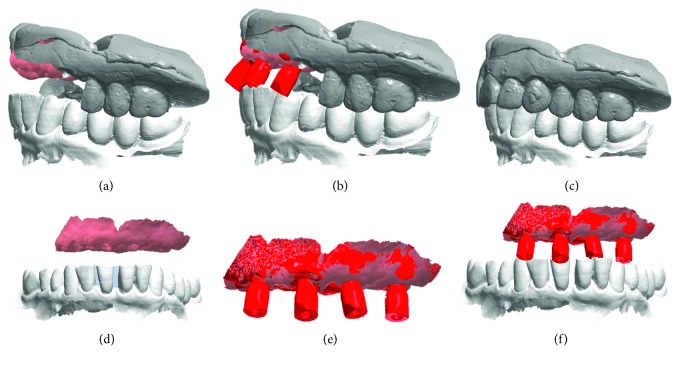
Intraoral scanning with CS 3600® (Carestream Dental, Atlanta, GE, USA), .STL files. The intraoral scan is performed with the patient wearing the copy of the preexisting denture, printed in 3D, properly discarded and opened in the scanbody area. The presence of this copy is essential to give the correct references for the vertical dimension of occlusion. (a) Master model with mucosal collars, antagonist, and copy of the preexisting denture opened in the anterior area; (b) master model with mucosal collars, antagonist, copy of the preexisting denture opened in the anterior area, and scanbodies; (c) copy of the preexisting denture and antagonist arch; (d) master model with mucosal collars and antagonist in the correct spatial relationship; (e) master model with mucous collars and scanbodies; (f) master model with mucosal collars, scanbodies, and antagonist in the correct spatial relationship.

**Figure 5:**
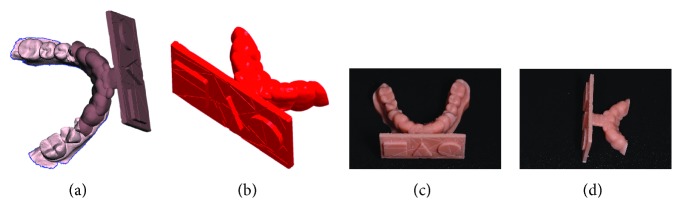
Designing and 3D printing of the individual reference tray (IRT), useful for the superimposition between intraoral scans and face scans. (a) IRT in Meshmixer® and its spatial relationship with the antagonist model; (b) detail of the IRT with known geometry; (c) printing of the tray and the model of the antagonist assembled together; (d) detail of the individual reference tray (IRT).

The surgery took place under local anesthesia, as previously described [29], by raising a full thickness flap and inserting 4 implants in the anterior area of the maxilla. The tapered implants used in this study (BTSafe®, BTK, Dueville, Vicenza, Italy) were characterized by double-lead threads with a hexagonal conical connection (11°) and integrated platform switching [33]. The dual acid-etched surface of these implants was the result of treatment with a strong inorganic acid mixture (H_2_SO_4_, H_3_PO_4_, HCl, and HF), giving the following roughness parameters: Ra = 1.12 (60.41) *μ*m, Rq = 1.34 (60.69) *μ*m, and Rt = 3.86 (61.40) *μ*m [34]. The implants were available in different diameters (3.3, 3.75, 4.1, and 4.8 mm) and lengths (8, 10, 12, and 14 mm). Once the implants were inserted and the sutures placed, the preexisting denture was discharged abundantly in the area of the implants (to avoid overloading), relined, and functionalized. The preexisting denture was carefully relined after functionalization and was therefore extraorally scanned with a structured light IOS (CS 3600®, Carestream Dental, Atlanta, Georgia, USA). Care was taken to capture the entire body of the denture (Figures 1(a)–1(f)) and, with it, the indirect functionalized impression of all the mucosal tissues, up to the area of the fornix and muscle insertions. The .STL file of the preexisting, relined, and functionalized complete denture was then imported into a free CAD software (Meshmixer®, Autodesk, San Rafael, CA, USA), where it was prepared for printing. Then, a replica of the preexisting relined and functionalized denture was 3D printed in a proprietary opaque resin (PrecisaRD097®, DWS, Thiene, Vicenza, Italy) using a stereolithographic (SLA) 3D printer (3500PD®, DWS, Thiene, Vicenza, Italy) (Figures 2(a)–2(c)). This replica was manually opened and discarded in the anterior area, corresponding to the implant scanbodies (Figures 2(d) and 2(e)). One week later, the patient was recalled for a second appointment, in which intraoral scans were taken with the aforementioned structured light IOS. The intraoral scan was performed using the dedicated implant acquisition mode (Figures 3(a)–3(c)). The clinician used a zig-zag technique: he started from the buccal side, carried occlusal and then palatal, and then returned to the occlusal, progressing constantly. The movement described by the tip of the scanner was therefore an arc, moving slowly to fly over the teeth and scanbodies, capturing all details possible but only in the area of interest. The scan started with the antagonist arch; then the master model was scanned, in order to capture the mucosal collars of the implants after the removal of the healing screws. The master model scan was performed with the patient wearing the copy of the preexisting removable denture, properly opened/discarded in the anterior area, i.e., the area of the implants. In other words, the mucosal collars and the soft tissues of the anterior area were visible and captured, but at the same time, the presence of the copy of the preexisting removable denture allowed the capture of the bite (occlusion). By capturing the bite, it was possible to get adequate information on the original vertical dimension of occlusion of the patient, given by the preexisting removable denture. After the capture of the bite, the mucosal collars were selectively cancelled, using the dedicated tools of the scanner acquisition software, and the scanbodies were screwed onto the implants. Thus, the first scan was completed with the capture of all the scanbodies in position. In this scan too, the patient had the replica of the denture in-mouth. Finally, before discharging the patient, since in this work the prosthetic bases were manufactured analogically, alginate impressions were recorded for the preparation of the individual tray useful for precision impressions and for the preparation of the prosthetic wax try-ins, for registering the vertical dimension of occlusion. After this meeting, all .STL files derived from the intraoral scan were saved in a dedicated folder, in the correct reciprocal spatial position (Figure 4); then, the scan of the preexisting denture of the patient was aligned on the master intraoral scan without scanbodies, using the teeth as reference points, via reverse engineering software (Studio 2012®, Geomagic, Morrisville, NC, USA). The files were then ready to be imported into Meshmixer®. Within this software, the model file of the opposing arch was used as basis for designing and modeling of the individual reference tray (IRT). The IRT was a bite splint modeled on the anterior teeth of the antagonist arch and therefore individualized; it was designed to fit firmly on the patient's antagonist model; an extraoral reference plate was therefore connected to this bite splint. This plate had geometric shapes (square, triangle, and circle) of known dimensions and was provided free of charge by the manufacturer of the powerful face scanner (OBI®, Fifthingenium, Milan, Italy) later used in this protocol, as an essential component in the process of superimposition between face scans and intraoral scans. Within Meshmixer®, through a few simple steps, the clinician modeled this individualized bite splint and “attached” it to the extraoral reference plate, obtaining the IRT (Figures 5(a) and 5(b)). The IRT was correctly positioned on the antagonist model, and all the models were in the correct spatial relationship to each other. The .STL files of the models were saved in a dedicated folder, and the IRT file was ready for 3D printing. The IRT was printed with the aforementioned 3500PD® SLA 3D printer, using the same proprietary opaque resin of the denture replica (Figure 5(c)).

**Figure 6:**
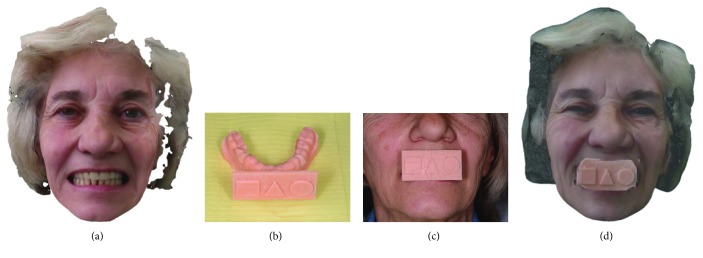
Face scan with OBI® (Fifthingenium, Milan, Italy), performed with the patient wearing a preexisting denture. (a) Face scan without an individual reference tray (IRT); (b) the individual reference tray is ready to be used; (c) extraoral detail of the individual reference tray (IRT) worn by the patient; (d) face scan with OBI® and individual reference tray (IRT).

**Figure 7:**
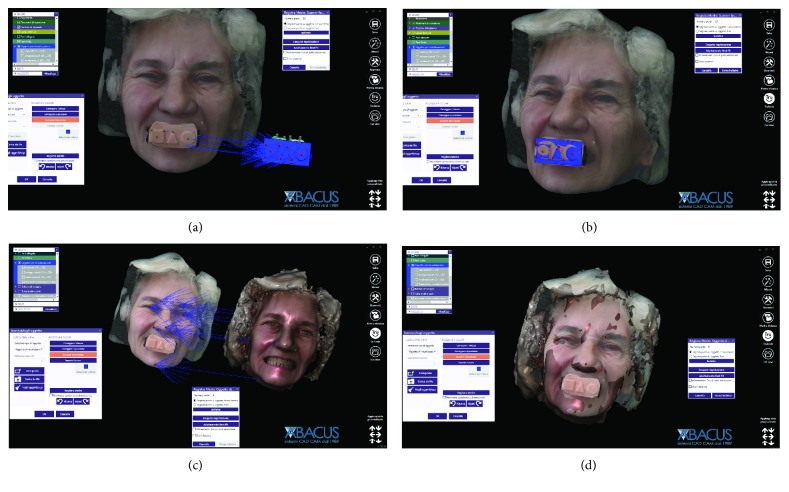
Import of all files from intraoral scan and face scan into the CAD software (Exocad®), in order to design the bar. (a) Import of face scan with individual reference tray (IRT); (b) superimposition by points and by surfaces of the face scan with individual reference tray (IRT) on the intraoral scan files, using the original CAD drawing of the tray; (c) import of the face scan without individual reference tray (IRT) and its superposition, by points and by surfaces, on the previous face scan, using facial landmarks; (d) when the superimposition is completed, it is now possible to design the bar having the morphology of the patient's face in the correct spatial position, without individual reference tray (IRT).

**Figure 8:**
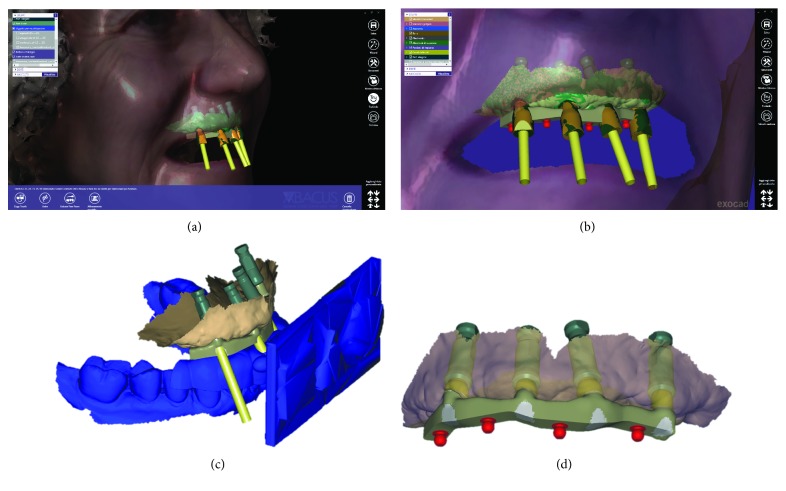
Design of the bar with the face references. (a) Detail of the modeled bar and scanbodies; (b) the bar modeled with precision attachments; (c) all the files are perfectly aligned within the CAD; (d) files of the final modeling of the bar.

**Figure 9:**
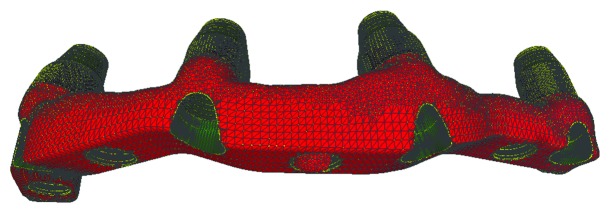
The design of the bar is ready for prototyping.

**Figure 10:**
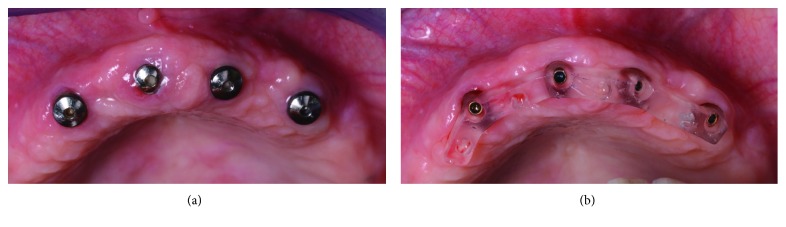
Test of the passive fit of the 3D-printed bar. (a) Healing abutments before removal; (b) the test of the 3D-printed bar in hard and transparent resin; it is essential to obtain a perfect fit on the implants and a passive fit.

**Figure 11:**
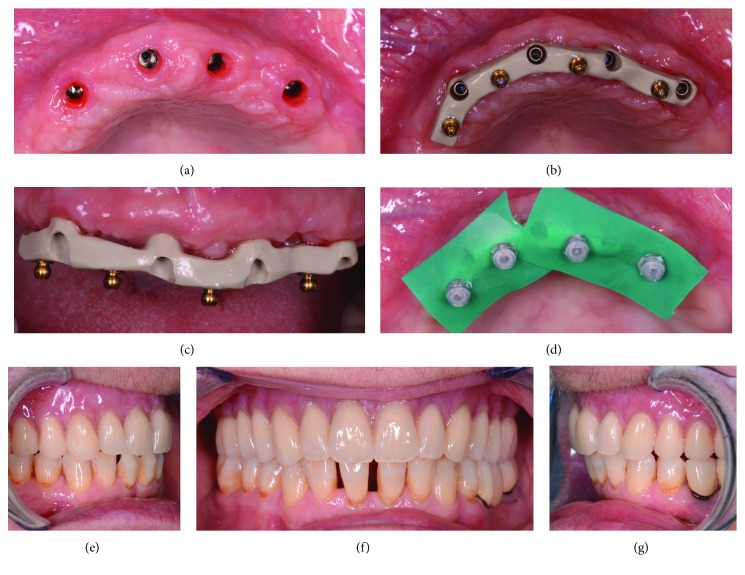
Delivery of the bar and the final overdenture. (a) Removal of healing abutments; (b) definitive PEEK bar, occlusal view; (c) definitive PEEK bar, front view; (d) activation of the prosthesis ball attachments directly in the mouth; (e) definitive overdenture, right side; (f) definitive overdenture, frontal view; (g) definitive overdenture, left side.

Once the IRT was ready, it was possible to recall the patient for the third appointment, in order to take the face scans of the patient, using the aforementioned face scanner (OBI®). The first face scan was captured with the smiling patient, without the IRT (Figure 6(a)). The second face scan was always carried out with the smiling patient, but with the IRT (Figures 6(b)–6(d)). In all, the two face scans took only 5 minutes and were performed with the scanner fixed on a tripod, and the patient in front of it, performing head movements, was guided by the acquisition software (turn left, right, up, and down). Both face scans were saved in .OBJ format and were ready for import into the prosthetic CAD software. All files (antagonist and master with copy of the preexisting complete removable denture opened in the anterior area, the latter with and without the scanbodies) deriving from Meshmixer®, along with the file IRT, were imported into a prosthetic CAD (Dentalcad®, Exocad, Darmstadt, Germany) for the modeling of the implant-supported bar, in respect of the correct spaces and prosthetic volumes. All files were in the correct reciprocal spatial position. At this point, the dental technician imported the face scans. The first face scan to be imported was the one with the IRT. This color texture, in .OBJ format, was superimposed on the CAD drawing of the IRT; the overlap took place first by points, using the geometric references of the tray (Figures 7(a) and 7(b)), and then by surfaces, in order to obtain an ideal alignment. The moving object was, of course, the face scan. Immediately after, the second face scan (without IRT) was also imported. This scan was therefore aligned on the previous face scan, using the same method described above. The overlap by points was performed using stable morphometric landmarks (pupils, tip and wings of the nose, eyebrows, and tip of the chin) and was therefore perfected by the automatic superimposition algorithm (Figures 7(c) and 7(d)). At this point, the face scan of the patient without the tray was perfectly aligned with the models and the dental technician could model the bar having all the information useful for the project: master model with mucosal collars and scanbodies, antagonist, and face scan. The face scan could be eventually cut out in the smile area, in order to provide more details regarding the positioning of the underlying prosthetic components (Figures 8(a) and 8(b)). The dental technician proceeded to replace the meshes of the implant scanbodies with the corresponding library files and modeled the implant-supported maxillary bar (Figures 8(c) and 8(d)). In the present study, the implants inserted had a complete and integrated library that allowed rapid CAD modeling in the correct positions. The customized CAD/CAM bar was anatomically designed by an experienced dental technician according to the implant position and the shape and volume of the preexisting removable complete denture, taking into account the information obtained with the face scans. Four precision attachments (spheres) were planned along the implant bar. The .STL file of the bar (Figure 9) was then exported and printed in 3D with 3500PD® using a proprietary transparent resin (DS300®), in order to obtain a replica of the bar, useful for checking the intraoral passivity and fit of the structure. This bar was tested in the patient's mouth, to check the adaptation, precision, and passive fit of the structure. For testing, it was screwed on all four implants to verify the passive fit (Figures 10(a) and 10(b)). Then, the bar was unscrewed and the functional tray was relined in the patient's mouth using a dedicated impression material (Permlastic®, Kerr, Orange, CA, USA). The bar remained included in this impression. Moreover, the vertical dimension of occlusion was recorded by means of the wax try-ins. The lab poured a master cast and manufactured a wax copy of the final denture, mounted in an articulator, for the aesthetic and functional tests. When the quality of the test bar had been verified, and the functional and aesthetic tests were performed with the wax copy of the final denture, it was possible to proceed with the manufacture of the definitive bar in polyether-ether-ketone (PEEK). The bar was manufactured from a block of PEEK in a milling center using a 5-axis milling machine (DWX-51®, Roland EasyShape, Ascoli Piceno, Italy). The dental technician polished the bar and cemented the ball attachments, so the definitive bar could be tested in the mouth. Again, passive adaptation of the structure and closures was verified clinically, before and after screwing. The final PEEK bar was delivered (Figures 11(a)–11(c)) and screwed on the implants, together with the final denture (Figures 11(d)–11(f)). The occlusion and the aesthetic integration were carefully verified. The patients were enrolled in a standard implant recall program. Oral hygiene maintenance was checked and radiographs were taken 1 year after the implant placement.

### 2.3. Clinical Outcome Measures

The outcomes of the study were the adaptation/passive fit of the bar on the implants, the functional/aesthetic integration of the overdenture, the 1-year implant survival, and the success rates of the implant-supported overdenture.

#### 2.3.1. Adaptation and Passive Fit of the Bar

The adaptation and passive fit of the bar were checked clinically, before and after screwing the replica (and the final bar) on the implants. The adaptation and passive fit were defined acceptable, in the absence of any movement of the bar before screwing, and when the bar was seated perfectly on the implants without any noticeable discrepancy. No difficulties were encountered when screwing the bar. In the case of movements of the bar during seating, or given evidence of discrepancies that could render the screwing on the implants difficult, the adaptation and passive fit were defined unacceptable, and so a new digital impression of the position of the implants, with and without scanbodies, had to be captured, in order to investigate the presence of any potential error(s) with the previous scan.

#### 2.3.2. One-Year Implant Survival Rate

Implant mobility in the absence of clinical signs of infection, nontreatable peri-implant infection (with pain, suppuration, and bone loss), severe progressive marginal bone loss in the absence of infection, and implant body fracture were the conditions for which an implant could be removed and consequently defined as “failed.” A distinction was made between “early” (within 3 months after implant placement) and “late” (at least 3 months after implant placement) failures. The 1-year implant survival rate was therefore calculated as the percentage of implant survival one year after placement. The implant survival rate was calculated at the patient level.

#### 2.3.3. One-Year Success Rate of the Implant-Supported Overdenture

In the absence of any biologic and prosthetic complications throughout the follow-up period, the implant-supported overdenture was considered successful. Biologic complications would include soft tissue inflammation (peri-implant mucositis) and peri-implant infection (peri-implantitis) with fistula formation, pain, and exudation/suppuration. The threshold for peri-implantitis was set by a probing pocket depth ≥ 6 mm with bleeding on probing and/or pus secretion. Prosthetic complications would encompass mechanical problems (loosening of the bar) and technical issues related to anchorage structure (broken bars or loose, lost, or broken attachments) or prostheses (repairs of fractured prostheses or overdenture teeth). The success rate of the overdenture was calculated at the patient level.

### 2.4. Statistical Evaluation

All data was collected from the records of the patients consecutively enrolled in the study. Descriptive statistics were performed for the patients' demographics (gender, age at start of the prosthetic treatment) and the diameter/length of the implants. Absolute and relative (%) distributions were calculated for qualitative variables (adaptation and passive fit, survival, and success rates). Finally, means, standard deviations, medians, and 95% confidence interval (95% CI) were estimated for quantitative variables (patient's age at start of the prosthetic treatment).

## 3. Results

The present clinical study was based on a sample of 15 patients (6 males, 9 females, mean age 68.8 ± 4.7 years, range 58–76, median 69, 95% CI: 66.5–71.1) rehabilitated with an implant-retained bar-supported maxillary overdenture. In all patients, the bar was fabricated in PEEK by means of a CAD/CAM procedure and was supported by 4 implants; thus, a total of 60 implants were placed. The distribution of the implants was as reported in Table 1.

At the time of testing the 3D-printed resin bar, 12 bars out of 15 (12/15: 80%) had a perfect passive adaptation and fit and were consequently considered acceptable; the technician could then proceed to mill the definitive PEEK bars. In contrast, 3 out of 15 resin bars (3/15: 20%) did not present a sufficient passive fit or adaptation, due to the presence of movements before screwing or difficulty in the screwing itself. In all these cases, it was therefore necessary to repeat the scanning, modeling, and production procedure. The repetition of the procedure allowed us to solve the problems and proceed with the manufacture of the final PEEK bars in a completely digital flow. At the time of the test and the delivery of the PEEK bars, on the contrary, no problem occurred. All the PEEK bars fit and screwed perfectly with an ideal passive fit and could therefore be safely delivered to the patient.

**Figure 12:**
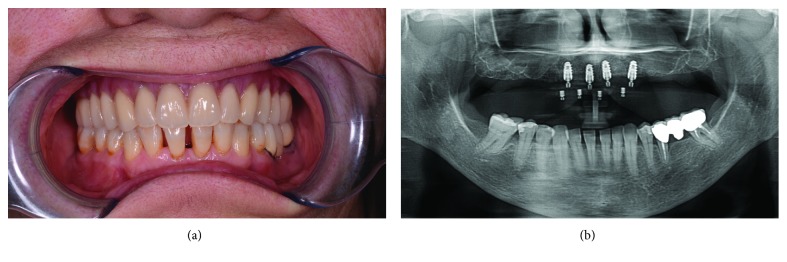
Clinical and radiographic control at 1 year from implant placement. (a) Frontal clinical photo; (b) panoramic radiograph.

No implants were lost, for a 1-year implant survival rate of 100% (60/60 surviving implants) (Figure 12).

Conversely, some complications (two fixtures with peri-implantitis, in the same patient; and two repaired overdentures because of tooth fracture, in two different patients) occurred during the follow-up period. This determined a 1-year success rate of 80% (12/15 patients without any complications encountered during the entire follow-up).

## 4. Discussion

The use of IOS for capturing optical impressions on natural teeth and on implants is rapidly spreading in dental offices around the world. The process of taking optical impressions is now comfortable for the patient [2, 20, 21] and capturing them is now easy for the clinician; at the same time, IOSs are accurate, as demonstrated by several *in vitro* studies [2, 3, 35], and allow the modeling of simple to complex fixed restorations that have a minimal marginal gap, as shown by several clinical studies [11–13, 16–19].

To date, the literature has not yet clarified whether optical impressions are able to capture quality impressions in the completely edentulous patient, both for fixed rehabilitations on implants and for the manufacture of removable implant-supported overdentures [22–26].

Despite this, the impressive technological evolution and the improvements in the acquisition software for IOS, with consequent enhancement of accuracy, open up new vistas and make it possible to extend the clinical applications of these instruments today, even to the completely edentulous patient.

In a recent clinical study, Capparè et al. [36] compared the accuracy of digital versus conventional impressions in the totally edentulous maxilla. In all, 50 patients who needed to be rehabilitated with full-arch Toronto screw-retained prostheses, each supported by 6 implants, were allocated to one of two groups: the *test* group (optical impressions with IOS) and the *control* group (conventional impressions) [36]. In the patients of the *test* group, the definitive metal structure of the prosthesis was milled in CAD/CAM, while in the patients of the *control* group, it was carried out in a conventional way [36]. In both groups, the passive fit and the marginal adaptation of the definitive structure were optimal, as also confirmed radiographically by the analysis of all 300 implants inserted; however, the digital procedure saved a great deal of time in the fabrication of the prosthetic structure [36]. The authors concluded that IOS represents a valid alternative for capturing suitable impressions for the modeling and fabrication of milled bars or structures, in support of full-arch prostheses in the maxilla [36].

This work has the merit of having highlighted how IOS is reliable and accurate in capturing the impression in the completely edentulous maxilla, confirming, in a larger sample of patients, the evidence that emerged in a previous work by the same authors [30]. It should be noted that all the scans were in the maxilla edentula, which is simpler than the edentulous mandible; furthermore, in this study, the implant scanbodies were splinted with resin [36].

Tallarico et al. [37] presented a protocol for the fabrication of overdentures, based on the extraoral chairside digitalization of scan abutments fixed on a specially designed customized tray, based on the original virtual planning. This custom tray allows one to reduce the error involved in intraoral scanning, providing landmarks to the scanner, and thus represents a valid alternative to splinting with resin; moreover, it allows the acquisition of information related to the occlusion register and the vertical dimension of occlusion, which are fundamental not only for the design of the bar but also for the design of the entire prosthesis in CAD/CAM [37]. In this sense, the use of face scanning can certainly help, in order to provide the technician with the information necessary for modeling, based on the information on the patient's face [37]. The construction of the overdenture in CAD/CAM, as well as that of the complete denture, starting with intraoral scanning, is essentially burdened by two practical problems: (a) the need to capture the scans of the arches in the correct vertical dimension of occlusion and thus in the proper spatial relationships and (b) the need (especially with the conventional removable denture) to obtain impressions that are correctly functionalized [38, 39]. Functionalization means the ability to record all the details of muscle insertions and frenula also in activity, which has always been a key in the making of a complete denture [38, 39]. As one might guess, it is very difficult if not impossible to capture optical impressions with IOS that are functionalized; the IOS, by definition, cannot capture dynamic changes in the soft tissues [38]. Precisely for this reason, the authors of previous studies on the fabrication of full digital removable dentures have always introduced analogic passages within the workflow, precisely because of the need to functionalize [38].

In the present prospective clinical study, 15 patients were enrolled and were rehabilitated with a maxillary bar-retained overdenture. The choice of an overdenture-type restoration (rather than a fixed restoration without fake gingiva) depended in this work on the absence of adequate facial support, as well as on economic (reduced cost) and hygienic reasons (ease of maintaining oral hygiene domiciliary, compared to Toronto fixed and screwed on the implants). The merit of this work was to present a technique for CAD/CAM fabrication of implant-supported bars for overdentures, starting with intraoral scanning. In this study, most of the CAD/CAM bars (80%) had an excellent passive fit and adaptation, with only a limited number of bars (3/15: 20%), which presented problems of fit and adaptation during the resin test. Although this percentage is rather high, representing about one bar out of five, it must be said that the repetition of the intraoral scan and the new design made it possible to overcome the problems and thus to create new test bars, which fitted perfectly on the implants. The passage through a test bar, 3D printed in resin, seems in this sense essential, before being able to pass to the production of the definitive PEEK bar, which obviously presents higher costs. Note that in all three cases of inadequate adaptation, the distal implants were rather tilted and disparallel to each other. These results seem to confirm the evidence emerged from the most recent studies, which show how the evolution of the software of IOS allows us today to capture sufficiently accurate impressions to support the fabrication of full-arch-type fixed prostheses [30, 31, 36, 37], with at least 4–6 implants. Of course, the accuracy of intraoral scanning depends on many factors, including the scanner used (different scanners give significantly different results) [35], the scanning strategy [40], and the operator's experience. The intraoral scanning strategy is certainly relevant, as different paths can determine different results [40]. In the present study, we have used a zig-zag technique, with the tip describing an arc over the surface of the teeth and scanbodies. This scanning path was selected because it gave excellent results in previous in vitro studies [3, 35]. In this work, the definitive CAD/CAM bars have been milled in PEEK. This choice is perfectly in tune with the metal-free philosophy, which is growing in digital dentistry; however, clinical studies are still needed to assess the performance and reliability of this material over the medium and long term [41]. In fact, although in this study all implants survived for one year, for a 100% survival rate, it should be noted that complications that were recorded during this study (two fixtures with peri-implantitis in the same patient and two repaired overdentures because of tooth fracture in two different patients) determined a success rate for the implant-prosthetic rehabilitation with overdenture of 80%, at 1 year. These complications must be taken into account, and the behaviour of soft tissues in relation to the PEEK of the bar should be further investigated.

This study has limitations: the low number of patients enrolled in the study, the limited follow-up, and the fact that only the bars (and not the prostheses) were manufactured in CAD/CAM. The limited follow-up is a particularly significant limitation of the present study, since we have used a relatively new material (PEEK) for the manufacture of the bars, which are normally made in metal. There are no long-term studies on the performance of PEEK bars and certainly an evaluation of at least 5 years is required, in order to draw adequate conclusions on the reliability of this method. Moreover, in this study, only the bars were CAD/CAM. The next step is undoubtedly represented by the possibility of using intraoral scans for the design and production of the overdenture prostheses themselves (and not just of the bars). This is technically possible today, using the setting and the acquisition protocol used in the present clinical study. The possibility of using the patient's face scans and working with the files of the prosthetic bases in the correct respective spatial positions, in full compliance with the vertical dimension of occlusion, represents a further merit of this study. The face scan is able to provide information on the patient's face, in 3D, to the dental technician; this information is very useful for modeling not only the bar with the relative dimensions but also and above all the removable overdenture, in full compliance with the tissue volumes [4, 5, 8]. The production of the final prosthesis can then rest on the milling of the pink acrylic prosthetic base and the teeth (which will be glued on top of it), as on 3D printing. Finally, a further limitation of the technique presented in this study is given by the costs of the machines (intraoral and face scanners, 3D printer) and necessary CAD software. The cost of these tools and software is still quite high, and this could limit the spread of the technique, making it not easily accessible to everyone. However, today, many dental practices invest in digital technologies, and it is not even necessary to buy everything: it is possible to rely on one of the many service centers (adequately equipped dental laboratories), at least for CAD software and 3D printers. In any case, when the whole process takes place within the dental clinic, in addition to the investment necessary for the purchase of devices and software, it is also necessary to consider that a learning curve is necessary, in order to learn how to use the machines and software. Digital processes are not simple, and this may represent a further limitation of the present study.

## 5. Conclusion

In the present clinical study, the integration of intraoral and face scans allowed us to successfully restore 15 fully edentulous maxillae maxillary overdentures supported by 4 implants and a CAD/CAM polyether-ether-ketone (PEEK) bar. In fact, when testing the 3D-printed resin bars (replicas), 12 bars out of 15 (12/15: 80%) had a perfect passive adaptation and fit and were consequently considered acceptable, i.e., the technician could proceed to mill the definitive PEEK bars. In contrast, three out of 15 bars (3/15: 20%) did not present a sufficient passive fit or adaptation, due to the presence of movements before screwing or difficulty in the screwing itself. In all these cases, it was necessary to repeat the scanning, modeling, and production procedure. The repetition of the procedure, however, allowed to solve the problems and proceed with the manufacture of the final PEEK bars in a completely digital flow. A 100% implant survival rate was found in this study; however, some complications (two fixtures with peri-implantitis in the same patient and two repaired overdentures because of tooth fracture in two different patients) occurred during the follow-up period, for a success rate of 80% for the implant-supported overdenture treatment. The digital procedures have the potential to decrease patient discomfort and to reduce the laboratory work associated with the fabrication of implant-supported overdentures. In addition, the use of PEEK can eliminate the need of using metals for the fabrication of the bar. However, this study has limitations, and further investigation is needed to confirm the outcomes emerging from this research.

## Figures and Tables

**Table 1:** Distribution of the implants (BTSafe®, BTK, Dueville, Vicenza, Italy) by length and diameter (in mm).

	8 mm	10 mm	12 mm	14 mm	Total
3.3 mm	7	8	5	2	22
3.75 mm	4	6	4	3	17
4.1 mm	5	4	6	1	16
4.8 mm	2	2	1	0	5
Total	18	20	16	6	60

## Data Availability

Data are available from the corresponding author upon reasonable request.
